# Spanish parents' emotion talk and their children's understanding of emotion

**DOI:** 10.3389/fpsyg.2013.00670

**Published:** 2013-09-24

**Authors:** Ana Aznar, Harriet R. Tenenbaum

**Affiliations:** ^1^Faculty of Arts and Social Sciences, Kingston UniversityLondon, UK; ^2^School of Psychology, University of SurreyGuildford, UK

**Keywords:** parent-child emotion talk, children's understanding of emotions, emotion understanding, culture, children's socialization of emotions

## Abstract

Relations between parent-child emotion talk and children's emotion understanding were examined in 63 Spanish mothers and fathers and their 4- (*M* = 53.35 months, *SD* = 3.86) and 6-year-old (*M* = 76.62 months, *SD* = 3.91) children. Parent-child emotion talk was analyzed during two storytelling tasks: a play-related storytelling task and a reminiscence task (conversation about past experiences). Children's emotion understanding was assessed twice through a standardized test of emotion comprehension (TEC; Pons et al., 2004), once before one of the two parent-child storytelling sessions and again 6 months later. Mothers' use of emotion labels during the play-related storytelling task predicted children's emotion understanding after controlling for children's previous emotion understanding. Whereas fathers' use of emotion labels during the play-related storytelling task was correlated with children's emotion understanding, it did not predict children's emotion understanding after controlling for previous emotion understanding. Implications of these findings for future research on children's socioemotional development are discussed.

## Spanish parent-child emotion talk and children's understanding of emotions

Emotion understanding is the ability to recognize, label, interpret, and respond to our own and others' emotions. Children's emotion understanding has been found to be an early predictor of later social adaptation (Izard et al., 2001), children's peer acceptance and popularity, prosocial behavior, and emotion regulation skills (Denham et al., 1990; Cassidy et al., 1992; Garner et al., 1994; Garner and Power, 1996; Hoffman, 2000). Children who have difficulty understanding and regulating their emotions have problems in their social relations with others (Denham et al., 1990; Kim and Cicchetti, 2010) and are likely to display long-term behavioral problems (Rydell et al., 2003). Children's understanding of emotions has also been linked to academic performance (Izard et al., 2001; Garner, 2010; Valiente et al., 2012), as well as to the development of psychopathology (Cicchetti et al., 1995; Kring and Bachorowski, 1999; Suveg et al., 2011).

Three broad levels of children's cognitive understanding of emotions have been identified (Pons et al., 2004). First, when children are 3 years of age they understand external aspects of emotion such as its situational causes (e.g., different situations provoke individuals to experience different emotions), its outward expression (e.g., individuals tend to express their emotions), and reminders' effect on affect (e.g., when someone reminds a child about a recently deceased pet, the child might experience sadness again). Children typically master this level of understanding by five. Between 5 and 7 years of age, children's understanding of emotions changes from a situational understanding to a mentalistic understanding of emotions (Wellman et al., 2001). They learn to recognize hidden emotions (Harris et al., 1986; Joshi and MacLean, 1994), and to understand the relationship between desires, beliefs and emotions (Harris et al., 1989). Finally, when children are between 7 and 11 years of age, they can reflect on an emotion from different perspectives. Children can understand ambivalent emotions, identify moral emotions, and are able regulate their emotions cognitively (Pons et al., 2004).

Despite evidence for a developmental pattern in children's emotion understanding, there are individual differences in children's emotion understanding from a very early age (Pons and Harris, 2005). Factors that underlie individual differences in children's emotion understanding have been investigated. During early infancy, parent-child emotion talk (Denham et al., 2000) and the emotional climate in the family (Denham et al., 2007; Zahn-Waxler, 2010) have been found to influence children's emotion understanding. Parental emotional expressiveness, its intensity, and parents' reactions to their children's expression of emotions have also been identified as influencing factors on children's emotion understanding (Denham et al., 1994).

Special attention has been paid to mother-child emotion talk and its relation with children's emotion understanding. In general, research has focused on the frequency and type of emotion talk (Dunn et al., 1987; Martin and Green, 2005). Indeed, the degree to which a child understands emotions is influenced by the frequency that his or her mother talks to him or her about emotions (Dunn et al., 1987; Denham et al., 1994; Denham and Auerbach, 1995; Halberstadt et al., 1999; Harris et al., 2005; Laible and Song, 2006). For example, Dunn et al. (1991) concluded that 3-year-olds living in families where emotions were discussed more frequently obtained higher scores than their peers when judging someone else's emotions at age six. Finally, Denham (1997) found that teachers rated children whose parents talked about emotions frequently as cooperative, empathic, and prosocial.

Not only is the frequency of mother-child emotion talk important, its quality might also be related to children's emotion understanding (Fivush, 1998). Specifically, it has been suggested that emotion explanations (e.g., “I am sad because my dog died”) and labels (e.g., “I am sad”) may help children conceptualize emotions differently (Cervantes and Callanan, 1998; Garner et al., 2008). Generally speaking, maternal explanations predict emotion understanding (Denham et al., 1994; Wellman and Lagattuta, 2004). Perhaps explanations about emotions allow children to learn about causes and consequences of emotions.

Little is known about the relation between father-child emotion talk and children's understanding of emotions. One way to address this gap in our knowledge is to directly compare mothers and fathers. The Spanish culture is an ideal context because one might expect differences between mothers and fathers, unlike American or UK samples. There have been repeated calls for more research on fathers and children (Lamb, 2010) because increasing research suggests that fathers influence children's outcomes, such as academic achievement (Fletcher et al., 1995). The little research there is on fathers has tended to focus more descriptively on how fathers talk to children (Reese et al., 1996). More research is needed that specifically looks at the link between fathers' talk and children's socio-emotional development.

Despite these calls for more research, only one study (Denham et al., 2010) has investigated fathers' emotion talk and children's understanding of emotions. Denham et al. (2010) found that mothers and fathers of preschool children differed in their socialization of emotions. Specifically, mothers acted as preschoolers' emotional gatekeepers, whereas fathers acted as preschoolers' playmates. However, mothers' and fathers' emotion socialization was similar for preschool boys and girls. Thus, based on this study we hypothesized that there would be differences in emotion talk between mothers and fathers. There are important differences between the present research and Denham's. First, Denham et al. (2010) asked parents and children to complete a reminiscence task, but not a storytelling task. In contrast, the present study incorporated a reminiscence task as well as a storytelling task. The use of both tasks is informative in that mothers' and fathers' talk during different tasks influences children's emotion understanding (Laible, 2004). The present study focused on mothers' and fathers' use of emotion labels and explanations across two different storytelling tasks. We expected children whose parents mentioned a higher proportion of emotion labels to have a better understanding of emotions than children whose parents mentioned fewer emotion labels. Second, Denham's study was restricted to children in early childhood. In addition to including 4-year-old children, we included 6-year-old children who represent a transitional phase between early and middle childhood. Finally, Denham et al.'s study examined North American children, whereas the current study focused on Spanish children.

Research suggests that culture influences children's understanding of emotions (Dunsmore et al., 2009; Halberstadt and Lozada, 2011; Perez-Rivera and Dunsmore, 2011). According to the enculturation perspective, children learn to understand how others feel and think embedded in the particular culture in which they live (Bruner, 1990). Similarly, culture influences parents' beliefs systems about how to raise children and about how to socialize their children's emotions (Dibiase and Gunnoe, 2004). Thus, parents and children influence each other with cultural expectations dictating how and when it is acceptable to display emotions (Brody, 1999).

Spain is regarded as a traditional culture with highly differentiated gender roles. Indeed, although through recent decades a higher number of women have joined the work forces (in 2010, 41.6% of women in Spain worked outside their homes), women still are children's primary caregivers and take care of most of the domestic chores (Instituto de la Mujer, 2012). In sum, Spanish women (similar to many Southern European women) hold a nurturant role and are regarded as the emotional keepers of the family (Dibiase and Gunnoe, 2004). We hypothesized that if Spanish mothers and fathers hold different roles when raising their children, Spanish mothers and fathers might have a distinct influence in their children's understanding of emotions. Given the amount of time that Spanish mothers compared to fathers spend with their children, we expected that there would be stronger relations between mothers' than fathers' talk with children's emotion understanding.

### The present study

The present study focused on 4- and 6-year-old children because most research examining parent-child emotion talk and children's understanding of emotions has analyzed children up to the age of four (Dunn et al., 1991; Wang, 2001; Wellman et al., 2001; Martin and Green, 2005; Denham et al., 2010), leaving little research of children's emotion understanding after the age of four. Of special relevance is the analysis of 6-year-olds given that this is a transitional age between preschool and middle childhood. Research on children's emotion understanding during middle childhood is needed as it has been argued that this capacity is further developed through this age (Flavell et al., 1993; De Rosnay and Hughes, 2006).

Parent-child emotion talk was analyzed across two storytelling tasks: a play-related storytelling task and a reminiscence task. Storytelling is relevant because it is a cultural activity in which emotions, societal norms, and values are embedded (Fivush, 1989). We chose the play-related storytelling task because research indicates that play is a valuable setting to prompt discussion about emotions between parents and children (Dunn et al., 1987; Cervantes and Callanan, 1998; Perez-Rivera and Dunsmore, 2011). During play parents may guide children's beliefs and ideas about emotions and children can improve their understanding of emotions (Fivush, 1993). Indeed, stories serve as an important cultural tool for expressing socio-cognitive understanding of emotions and beliefs (Fivush, 1989). Moreover, it might be easier for children to discuss someone else's emotions rather than their own because they distance themselves to focus on a cognitive understanding of emotion. It has also been suggested that fictional narratives are a more controlled form of discourse than personal narratives (Bamberg and Damrad-Frye, 1991).

The reminiscence task was used because when reminiscing, parents and children not only discuss specific details of what happened but they also tend to discuss how those events made them feel (Fivush et al., 2009). Moreover, reminiscing gives children an opportunity to put emotions into perspective, which is more difficult when discussing present emotions (Fivush, 1989; Laible, 2004). Children's ability to elaborate on their personal narratives has been linked to their understanding of emotions (Cutting and Dunn, 1999). This study is the first to investigate parent-child emotion talk across these two types of task and relate it to children's emotion understanding. Based on previous research, we expected children of mothers and fathers who mentioned a higher proportion of emotion words to have a better understanding of emotions after controlling for prior emotion understanding. In addition, we expected that relations between mothers' talk and children's emotion understanding would be greater than that of fathers' talk and children's emotion understanding based on gender differences in Spanish culture.

## Methods

### Participants

Sixty-three children (30 girls and 33 boys), aged 4 (17 girls *M* = 53.20 months, *SD* = 3.86; range = 48–60 months; 18 boys, *M* = 53.21, *SD* = 3.88; range = 48.10–59.50 months) and 6-years-old (13 girls *M* = 76.79 months, *SD* = 3.92; range = 72–83.50 months; 15 boys, *M* = 76.21, *SD* = 3.86; range = 72–83.50 months) participated with both of their parents (mothers' age was *M* = 36.30 years, *SD* = 2.88; range = 29–42 years; fathers' age was *M* = 40.60 years, *SD* = 4.42; range = 34–54 years). The average number of children per family was 2.76 (*SD* = 0.95). Of the child participants, 24 were firstborns and the rest were later-borns. ANOVA models revealed no relations between number of siblings and children's birth order with emotion understanding or parental emotion talk.

All families were Spanish with Spanish as their first language. All families were intact from predominantly middle-to upper-middle class socioeconomic status. Parents had attended university. Participants were recruited on a volunteer basis. All parent signed an informed consent form. This study was part of a larger investigation of the relation between parent-child emotion talk, parent-child touch, and children's emotion understanding.

### Materials

#### Play-related storytelling task

A plastic house and six family figures which included a grandfather, a grandmother, a father, a mother, a son, a daughter and a dog were used for the play-related storytelling task. The house was divided into four rooms: a kitchen, a living room, a bedroom, and a bathroom.

#### Reminiscence task

To elicit talk about emotions, parent-child dyads were given four events typed individually on index cards. Each card contained one of the following sentences: “a visit to the zoo,” “a visit to the doctor,” “the first day of school,” and “a time that the child fell down.”

#### Test of emotion comprehension

A standardized Spanish version of the Test of Emotion Comprehension (TEC, Pons et al., 2004) was administered to the child participants. The TEC assesses emotion understanding of 3-to-11 year old children by presenting vignettes in which a gender-matched protagonist encounters simple to complex situations eliciting different emotional responses. After each vignette, the child is asked how the protagonist feels by choosing from four illustrations of faces representing different emotional states (happiness, sadness, fear, normal, or anger). Vignettes are organized in an increasing order of difficulty (Pons et al., 2004). The TEC asks the child to identify emotions in 9 different situations, namely: (1) represented by facial expressions, (2) caused by external situations, (3) involving situations in which emotions result from desires, (4) consequence of a character's false belief, (5) elicited by reminders, (6) when a character attempts to control an emotion, (7) hidden, (8) conflicting, and (9) stemming from self-restraint. To test for understanding of hidden emotions, the child is asked how a teased character feels although he/she is smiling. Understanding of conflicting emotions is tested by asking the child about how a character feels after receiving a new bicycle when the character has never ridden a bicycle and could fall.

The TEC was the chosen test to assess children's level of emotion understanding because it has been widely used and replicated. Its different components are scalable (index of consistency *I* = 0.676) and the scale is valid (Coefficient of reproducibility *R* = 0.904; Pons et al., 2002). In addition, the TEC is different from other tests of emotion comprehension in the simplicity of the language that it uses (Pons et al., 2003). This reduces the effect of language ability on the understanding of emotions. The TEC has a high test-retest correlation (*r* = 0.83) within a 3-month period (Pons et al., 2002) and a 13-month period (*r* = 0.68; Pons and Harris, 2005) of the TEC has been found. The TEC has been used with Spanish-speaking children (Jimenez et al., 2013).

### Procedure

Parent-child dyads were interviewed in their own homes on 2 separate days. Parents were informed that we were interested in parent-child interactions. On a first visit, the mother or the father and the child completed the play-related storytelling task and the reminiscence task. Within a minimum of one day and a maximum of 7 days, the other parent and the child completed the same two storytelling tasks. These two tasks were counterbalanced. Parent order was also counterbalanced. ANOVA models conducted on parents' speech variables revealed no effects of either parent or task order. The length of time devoted to the storytelling tasks was determined by participants as it has been argued (e.g., Kuebli and Fivush, 1992; Cervantes and Callanan, 1998; Fivush et al., 2000) that in this manner emotions are used in a more naturalistic manner. Mothers' conversations lasted for an average of 18.18 min (*SD* = 7.44) and fathers' conversations lasted for an average of 21.63 min (*SD* = 6.97). These sessions were videotaped.

In the play-related storytelling task, the first author asked the parent and the child to use the figures and the house to create a story together. To elicit the story, parent-child dyads were orally provided with four events: (1) the parents leave their children to go on an overnight trip, (2) the child falls down and hurts himself, (3) the dog runs away, and (4) the parents return home. This task has been very useful in eliciting discussion about emotions, and it has been used in a number of studies (e.g., Bretherton et al., 1990; Oppenheim et al., 1993; Cervantes and Callanan, 1998; Martin and Green, 2005). Events 1, 2, and 4 were taken from the attachment story-completion task by Bretherton et al. (1990). The four events have important emotional themes for preschool children (Cervantes and Callanan, 1998).

The other task was a reminiscence task. The first author gave parent-child dyads four events typed in four index cards: (i) the child's first day in school, (ii) a visit to the doctor, (iii) a time that the child fell down, and (iv) a trip to the zoo. Participants discussed the events in the order that they chose. These four events were used because of two reasons. First, they all involve important events for preschool children. Second, research suggests that not only do children's conversations about their own emotions play an important role in children's socialization of emotions, they may enhance children's understanding of emotions (Fivush, 1989).

In addition, the TEC was administered to the child participants before one of the two parent-child storytelling sessions and again 6 months later. All children completed the second TEC within a week of the 6-month mark. The TEC was administered in a quiet room in the presence of their parents. Its administration typically lasted 10 min.

### Transcription and coding

Videotaped conversations were transcribed verbatim by the first author and a native Spanish research assistant. The first author (a native Spanish speaker) coded all transcripts and the second author (a fluent Spanish speaker) checked them for accuracy. Transcripts were coded for mothers' and fathers' (1) total number of utterances, (2) total number of emotion utterances, and (3) emotion labels vs. emotion explanations.

#### Total number of utterances

The number of utterances made by the mother and the father were recorded. An utterance was a message unit bound by its intonation (Hoff-Ginsberg, 1991). Excluded were unintelligible utterances and false starts (Pancsofar and Vernon-Feagans, 2006). The first author typed transcripts into utterances, which were checked by the second author.

#### Total number of emotion words

Emotion words were those referring to a specific affective state (e.g., angry, jealous), or process (e.g., to have fun, to be furious, Cervantes, 2002). Total number of emotion words was calculated by adding together the total number of times each emotion word was used. The total number of emotion words mentioned by the mother and the father throughout the conversations were identified.

#### Emotion labels versus emotion explanations

All emotion words were coded as to whether they occurred in a label or explanation. Labels were emotion words that made reference to an emotion or ask about an emotion without including a causal relationship (e.g., “I am very happy”). Explanations were emotion references that asked about an emotion or that make a statement about an emotion including a causal relationship (e.g., “I am very happy because my mum gave me a present”). Emotion words were also coded as explanations if there was a causal link (e.g., “I am very happy because my mum gave me a present”), a lexical causative (e.g., “My mum made me very happy when she gave me a present) or if there was no explicit causal link but the utterances were adjacent and were rated as semantically causal (e.g., “I am very happy. My mum gave me a present”). These criteria are based on Bloom and Capatides (1987) and have been used in previous studies (Cervantes and Callanan, 1998; Martin and Green, 2005).

### Reliability

Intercoder reliability was established separately for each coding scheme. Each child participant had four transcripts: one for each task (play-related storytelling task and reminiscence task) with each of his or her parents. The first author coded all transcripts and the second author coded 46 transcripts (20% of the data set). Coding was conducted in Spanish. Coders were not blind to the research hypotheses. Reliability was achieved with a K of 0.80 for the total number of emotion words, and with a K of 0.91 for the total number of emotion labels and explanations.

### Scoring of the TEC

Following the standard TEC scoring procedure, children received a point for each of the nine components answered correctly, with a highest possible score of nine and lowest possible score of zero. Overall performance on the test was computed for each child by summing his or her scores on each of the nine emotion components. Children received credit when they indicated the correct emotion. The first two components, namely recognition and external cause, included five test items. Children received a single point on each of these components if they were correct on at least four of the five items. The third component, desire, included two test questions. Children received a single point if they were correct on both test items. The remaining six components included one test question. Children received one point for each emotion component on which they succeeded. Overall scores could range from zero to one on each component and from zero to nine on the entire test. Situational, mentalistic, and reflective levels of emotion were each based on three test questions added together.

## Results

Each child had four transcripts: mother-child play-related storytelling task, mother-child reminiscence task, father-child play-related storytelling task, and father-child reminiscing task. Only children who produced four transcripts were included in the analysis.

### Data analyses

First, we present descriptive statistics for parents' talk and children's TEC scores. Next, correlations between the variables of interest are presented. Finally, regressions predicting children's emotion understanding at the second time point are presented.

Previous studies have analyzed emotion talk as total frequencies or as proportions. In this study and similar to previous research (e.g., Garner, 2003; Sales et al., 2003; Curenton and Craig, 2011; Garrett-Peters et al., 2011; Brownell et al., 2013), emotion words were analyzed as proportions. By using proportions rather than total frequencies, participants' total amount of talk was controlled. Proportions were calculated as the total number of emotion utterances divided by the total number of utterances. These were calculated separately for each participant.

### Descriptive statistics

The majority of parents discussed emotions throughout both storytelling tasks even though they were not explicitly asked to discuss emotions. During the play-related storytelling task, mothers used a mean proportion of 0.32 emotion labels (*SD* = 0.30) and 0.21 explanations (*SD* = 0.22), while during the reminiscence task, mothers used a mean proportion of 0.98 emotion labels (*SD* = 0.76) and 0.12 explanations of (*SD* = 0.15).

During the play-related storytelling task, fathers used a mean proportion of 0.24 emotion labels (*SD* = 0.22), and 0.10 explanations (*SD* = 0.12). Finally, fathers used a mean proportion of 0.66 emotion labels (*SD* = 0.58), and 0.08 explanations (*SD* = 0.14) during the reminiscence task.

#### Descriptive analyses on TEC 1 and TEC 2

Across both age groups, the minimum score was two and the maximum score was eight. A 2 (Children's age: 4, 6) × 2 (Children's gender: girl, boy) analysis of variance (ANOVA) conducted on the first administration of the TEC 1 as a dependent variable revealed no significant effect of gender. As expected, it revealed a significant age effect with 6-year-old children scoring higher on the TEC (*M* = 6.53, *SD* = 0.99) than 4-year-old children (*M* = 4.48, *SD* = 1.44), *F*_(1, 62)_ = 42.35, *p* < 0.001, η^2^ = 0.41. A 2 (Children's age: 4, 6) × 2 (Children's gender: girl, boy) analysis of variance (ANOVA) conducted on the situational level of TEC 1 as a dependent variable revealed no significant effect of gender. As expected, it revealed a significant age effect with 6-year-olds scoring higher on the situational level of TEC 1 (*M* = 2.54, *SD* = 0.58) than 4-year-olds (*M* = 2.00, *SD* = 0.94), *F*_(1, 62)_ = 6.79, *p* = 0.01, η^2^ = 0.10. There was also a significant Age × Gender interaction effect, *F*_(1, 62)_ = 6.91, *p* = 0.01, η^2^ = 0.11. There was no difference between girls and boys at age 4, *F*_(1, 62)_ = 2.14, *p* = 0.15, whereas boys (*M* = 2.80, *SD* = 0.41) outperformed girls (*M* = 2.23, *SD* = 0.60) at age 6, *F*_(1, 27)_ = 8.75, *p* = 0.007, η^2^ = 0.25.

A 2 (Children's age: 4, 6) × 2 (Children's gender: girl, boy) analysis of variance (ANOVA) conducted on the mentalistic level of TEC 1 as a dependent variable revealed no significant effect of gender. As expected, it revealed a significant age effect with 6-year-olds scoring higher on the mentalistic level of TEC 1 (*M* = 2.25, *SD* = 0.64) than 4-year-olds (*M* = 1.45, *SD* = 1.01), *F*_(1, 62)_ = 12.65, *p* = 0.001, η^2^ = 0.18. Finally, a 2 (Children's age: 4, 6) × 2 (Children's gender: girl, boy) analysis of variance (ANOVA) conducted on the reflective level of TEC 1 as a dependent variable revealed no significant effect of gender. As expected, it revealed a significant age effect with 6-year-olds scoring higher on the reflective level of TEC 1 (*M* = 1.04, *SD* = 0.69) than 4-year-olds (*M* = 0.40, *SD* = 0.50), *F*_(1, 62)_ = 9.02, *p* = 0.004, η^2^ = 0.13. There was also a significant Age × Gender interaction effect, *F*_(1, 62)_ = 8.04, *p* = 0.006, η^2^ = 0.12. There was no difference between girls and boys at age 4, *F* < 1, whereas girls (*M* = 1.46, *SD* = 0.52) outperformed boys (*M* = 0.67, *SD* = 0.62) at age 6, *F*_(1, 27)_ = 13.36, *p* = 0.001, η^2^ = 0.34.

Similarly, a 2 (Children's age: 4, 6) × 2 (Children's gender: boy, girl) ANOVA conducted on the second administration of the TEC as a dependent variable revealed no significant effect of gender. There was, however, a significant age effect with 6-year-old children scoring higher on the TEC (*M* = 6.35, *SD* = 1.40) than 4-year-old children (*M* = 4.68, *SD* = 1.72), *F*_(1, 62)_ = 16.56, *p* < 0.001, η^2^ = 0.21. Scores for TEC 1 and TEC 2 were significantly correlated, *r*_(61)_ = 0.78, *p* = 0.01. A repeated measures ANOVA revealed that across both age groups, children scored higher on TEC 2 than on TEC 1, *F*_(1, 62)_ = 22.88, *p* < 0.001, η^2^ = 0.27.

### Relations between parents' emotion talk, and children's emotion understanding

Before conducting analyses, all data were screened. Children's TEC scores and mothers' emotion labels and explanation in each task were found to be normally distributed with kurtosis and skewness below 3.00 (Tabachnik and Fidell, 2007). In contrast, fathers' use of explanations during the storytelling task and explanations and labels during the reminiscence task were found to violate skewness and kurtosis assumptions. Removal of four outliers did not improve skewness and kurtosis to below 3.00. Thus, a square root transformation was applied to these variables in SPSS.

**Figure 1:**
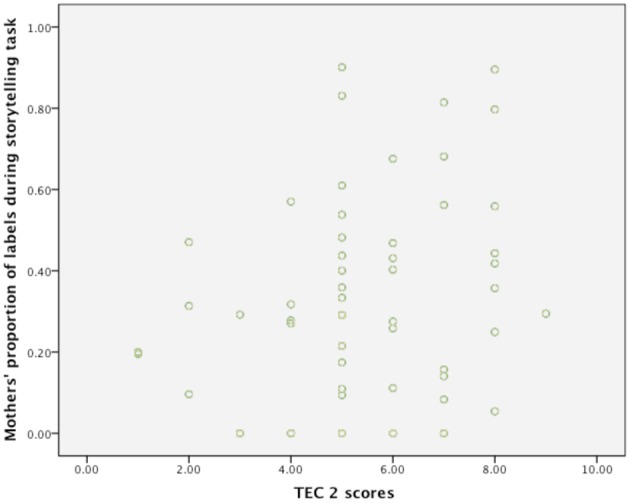
**Proportion of labels used by mothers during the storytelling task and TEC 2 scores**.

To examine which elements of mothers' and fathers' emotion talk were related to the TEC 1 and TEC 2, correlations were conducted between TEC 1 and TEC 2 and mothers' emotion talk (emotion labels and emotion explanations during the play-related storytelling task and the reminiscence task) and fathers' emotion talk (emotion labels and emotion explanations during the play-related storytelling task and the reminiscence task). Figure 1 indicates the relation between mothers' proportion of labels during the storytelling task and TEC 2 scores. These correlations were conducted separately for each group and combined across age groups using the transformed scores for three of the fathers' talk variables. Table 1 indicates that TEC1 and TEC 2 were highly correlated, *r*_(61)_ = 0.78, *p* = 0.01, across both age groups combined. There was a significant relation between mothers' labels during the reminiscence task and TEC 2, *r*_(61)_ = 0.28, *p* = 0.05. There was also a significant relation between fathers' labels during the reminiscence task (transformed variable) and TEC 2, *r*_(61)_ = 0.28, *p* = 0.05, and fathers' explanations (transformed variable) and labels during the reminiscence task, *r*_(61)_ = 0.27, *p* = 0.05. Fathers' use of labels during the two tasks was correlated, *r*_(61)_ = 0.37, *p* = 0.003. There was a significant correlation between mothers' explanations and labels during the storytelling task, *r*_(61)_ = 0.27, *p* = 0.05. Mothers' use of labels during both tasks was correlated, *r*_(61)_ = 0.38, *p* < 0.01. Table 2 displays the correlations conducted separately for 4-year-old children. In the case of 4-year-old children TEC 1 and TEC 2 were related, *r*_(61)_ = 0.73, *p* = 0.01. Table 3 displays the correlations conducted separately for 4-year-old children. There was a significant relation between 6-year-olds' TEC1 and TEC 2, *r*_(61)_ = 0.65, *p* = 0.01. No correlations between parents' emotion talk and children's emotion understanding were significant when the statistics were conducted separately by age group, however as the tables indicate, there were relations between parents' talk variables. For example, when children were 4-years-old, there was a significant relation between mothers' use of labels during the two tasks, *r*_(61)_ = 0.38, *p* < 0.05. For fathers, fathers' use of labels during the two tasks was significantly related, *r*_(61)_ = 0.37, *p* < 0.01. When children were 6-years-old, there was also a relation between mothers' use of labels across the two tasks, *r*_(61)_ = 0.43, *p* < 0.05.

**Table 1:** **Relations between parental talk and children's emotion understanding**.

	**1**	**2**	**3**	**4**	**5**	**6**	**7**	**8**	**9**	**10**
1. TEC 1	−									
2. TEC 2	0.78^**^									
3. Mothers' proportion of labels: storytelling task	0.005	0.2								
4. Mothers' proportion of explanations: storytelling task	−0.13	−0.06	0.27^*^							
5. Mothers' proportion of labels: reminiscence task	0.21	0.28^*^	0.38^**^	−0.002						
6. Mothers' proportion of explanations: reminiscence task	0.41	0.06	0.18	0.06	0.23					
7. Fathers' proportion of labels: storytelling task	0.24	0.28*	−0.05	−0.18	0.04	0.1				
8. Transformed fathers' proportion of explanations: storytelling task	−0.15	−0.03	0.05	−0.07	−0.11	−0.14	0.27^*^			
9. Transformed fathers' proportion of labels: reminiscence task	0.02	0.13	0.10	−09	0.21	−0.01	0.37^**^	0.31^**^		
10. Transformed fathers' proportion of explanations: reminiscence task	0.08	0.21	0.17	0.17	0.12	0.12	0.33^**^	0.32^**^	0.48^**^	−

** p ≤ 0.01;

* * p ≤ 0.05*.

**Table 2:** **Relations between parental emotion talk, and children's emotion understanding at age 4**.

	**1**	**2**	**3**	**4**	**5**	**6**	**7**	**8**	**9**	**10**
1. TEC 1										
2. TEC 2	0.73^**^									
3. Mothers' proportion of labels: storytelling task	0.04	0.22								
4. Mothers' proportion of explanations: storytelling task	−0.06	−0.01	0.22							
5. Mothers' proportion of labels: reminiscence task	0.14	0.24	0.38^*^	0.04						
6. Mothers' proportion of explanations: reminiscence task	0.07	0.14	0.37^*^	0.07	0.47^**^					
7. Fathers' proportion of labels: storytelling task	0.15	0.31	0.22	0.05	0.25	0.17				
8. Transformed fathers' proportion of explanations: storytelling task	−0.25	−0.27	−0.05	−0.17	−0.08	−0.02	0.28			
9. Transformed father's proportion of labels: reminiscence task	−0.04	0.13	0.14	0.04	0.30	0.16	0.44^**^	0.19		
10. transformed fathers' proportion of explanations: reminiscence task	−0.18	0.14	0.28	0.38^*^	−0.06	0.34^*^	0.35^*^	0.20	0.044^**^	−

** p ≤0.01;

* * p ≤ 0.05*.

**Table 3:** **Relations between parental emotion talk and children's emotion understanding at age 6**.

	**1**	**2**	**3**	**4**	**5**	**6**	**7**	**8**	**9**	**10**
1. TEC 1										
2. TEC 2	0.65^**^									
3. Mothers' proportion of labels: storytelling task	−0.16	0.2								
4. Mothers' proportion of explanations: storytelling task	−0.23	−0.1	0.40^*^							
5. Mothers' proportion of labels: reminiscence task	0.14	0.23	0.43^*^	−0.02						
6. Mothers' proportion of explanations: reminiscence task	−0.14	−0.16	−0.19	0.06	−0.03					
7. Fathers' proportion of labels: storytelling task	0.24	0.11	−0.51^**^	−0.46^*^	−0.2	−0.3				
8. Transformed fathers' proportion of explanations: storytelling task	−0.07	0.31	0.21	0.06	−0.14	−0.28	0.27			
9. Transformed fathers' proportion of labels: reminiscence task	−0.23	−0.06	0.01	−0.24	0.09	−0.27	0.25	0.46^*^		
10. Transformed fathers' proportion of explanations: reminiscence task	−0.08	0.02	−0.01	−0.24	0.18	−0.15	0.26	0.46^*^	0.48^*^	−

** p ≤ 0.01;

* * p ≤ 0.05*.

#### Regression analyses

To examine whether scores on TEC 2 were predicted by mothers' and fathers' emotion talk after controlling for prior TEC scores, two hierarchical multiple regression analyses were conducted separately for each parent with TEC 2 as the dependent variable. Regressions were conducted separately because of inadequate statistical power (Field, 2005). In step 1, TEC 1 was entered. In step 2, the parent's proportion of labels and explanations during the play-related storytelling task and the reminiscence task were entered. As expected, TEC 1 predicted TEC 2. Above and beyond prior emotion understanding, fathers' talk did not predict children's emotion understanding. In contrast, mothers' emotion labels during the play-related storytelling task predicted children's understanding of emotions. Tables 4, 5 display the beta weights and standard errors.

**Table 4:** **Regression model predicting children's emotion understanding from maternal talk**.

		**SE**		
		***B***	**B**	**B**
Step 1	Constant	1.51	0.43	
	TEC 1	0.83	0.09	0.78^**^
Step 2	Constant	1.15	0.48	
	TEC 1	0.81	0.09	0.76^**^
	Mothers' proportion of labels: story task	1.13	0.54	0.19^*^
	Mothers' proportion of explanations: story task	−0.11	0.67	−0.01
	Mothers' proportion of labels: reminiscence task	0.13	0.21	0.05
	Mothers' proportion of explanations: reminiscence task	−0.24	0.93	−0.02

*R^2^ = 0.60 for Step 1, ΔR^2^ = 0.05 for Step 2 (p < 0.05)*.

** p < 0.05;

* *p < 0.01*.

**Table 5:** **Regression model predicting children's emotion understanding from paternal talk**.

		**SE**		
		***B***	**b**	**B**
Step 1	Constant	1.52	0.43	
	TEC 1	0.83	0.09	0.78
Step 2	Constant	3.98	1.73	
	TEC 1	0.81	0.09	0.76
	Fathers' proportion of labels: story task	0.26	0.73	0.03
	Fathers' proportion of explanations story task	0.22	0.72	0.03
	Fathers' proportion of labels: reminiscence task	0.21	0.51	0.04
	Fathers' proportion of explanations: reminiscence task	0.90	0.73	0.12

*R^2^ = 0.61 for Step 1, ΔR^2^ = 0.03 for Step 2*.

## Discussion

This study explored the relation between Spanish parent-child emotion talk and children's emotion understanding across two types of family narratives: a play-related storytelling task and a reminiscence task. Findings of this study support and extend the existing research on parent-child emotion talk and children's emotion understanding. The correlations between mothers' and fathers' emotion talk and children's emotion understanding were similar. Mothers' use of emotion labels during the play-related storytelling task predicted children's emotion understanding above and beyond prior emotion understanding, whereas, fathers' emotion talk did not predict children's emotion understanding above and beyond prior emotion understanding. Thus, after controlling for prior emotion understanding, mothers' influence continues to be more predictive of children's understanding than is fathers' influence. These findings will be discussed in greater detail below.

Mothers' use of labels during the reminiscence task and fathers' use of labels during the play-related storytelling task was related to children's emotion understanding at time 2. However, after controlling for prior emotion understanding, fathers' emotion talk did not predict children's emotion understanding. This finding is not consistent with Denham et al. (2010) who concluded that fathers' emotion talk is related to children's emotion understanding. There are a few reasons that could explain the different findings between both studies. We conjecture that Spanish mothers' and fathers' influence on their children's development of emotion understanding is more distinct to that of US mothers and fathers. Indeed, evidence suggests that mothers are the emotional gatekeepers of the family whereas fathers occupy playmate and disciplinarian roles (Garside and Klimes-Dougan, 2002; Lewis and Lamb, 2003; Bretherton et al., 2005; Denham et al., 2010). Perhaps fathers socialize children's emotions through rough and tumble play or through the way in which they discipline their children. Indeed, fathers' play styles predict children's later socio-emotional development (Carson and Parke, 1996). Alternatively, fathers may have been following children's lead from lack of daily experience with them, and used more emotion words when their children afforded such talk. Another possibility is that we cannot study mothers' and fathers' socialization of emotions using the same methodology and instead, should develop a specific method to examine fathers' contribution to children's emotion understanding.

One final explanation is that fathers tend to spend less time with children than do mothers (Craig, 2006). In Spain, this gender difference is exacerbated with mothers as children's main careers (Instituto de la Mujer, 2012). Less than 20% of the mothers worked fulltime outside the home in this sample with many stay-at-home mothers. In contrast, the fathers tended to work long hours and many reported working more than 50 h a week. Thus, how much time fathers typically spent with children is unknown and may be one of the reasons why their influence was smaller.

Second, mothers' use of emotion labels during the play-related storytelling predicted children's emotion understanding. We conjecture that when mothers labeled emotions during the play-related storytelling task, they drew children's attention to them and implicitly communicated to children that expressing emotions is acceptable. Indeed, Denham et al. (2010) suggest that mothers serve as emotional gatekeepers of the family. For this reason, we find it striking that mothers' emotion talk during reminiscing did not predict children's emotion understanding. Perhaps during reminiscing the emotional experience is too strong for children to learn from the experience compared to the storytelling task. Note that the TEC is a cognitive measure of emotion understanding rather than an expressive measure. Moreover, like a storytelling task, the children need to understand emotions another person experiences to answer questions correctly on the TEC, which may help to explain why there is a relation between mothers' labels during the storytelling task and the TEC, but not during the reminiscence task. There may be other elements of mothers' emotion talk while reminiscing that predict children's emotion understanding, such as mothers' elaborateness or the discussion of causes and consequences of emotions.

Little research on parent-child emotion talk and its relations with children's emotion understanding has been conducted outside English-speaking countries or with fathers. This study demonstrates that we cannot simply extend the literature on mothers to fathers. Fathers have a distinct influence on children's development that must be investigated. Research needs to include fathers in more cross-cultural research to examine how their participation influences children.

### Limitations and directions for future research

The present study found some small relations between parents' emotion talk and children's emotion understanding; however, many of the correlations were not significant. Thus, we need to be cautious when interpreting the findings of this study. Moreover, when the correlations were conducted separately by age group, there were no significant relations between parental talk and children's emotion understanding. The lack of findings may have resulted from the small sample size when the ages were split or resulted from other factors influencing children's emotion understanding. Future research should examine other factors such as children's language abilities, given that language ability influence children's emotion understanding (Harris et al., 2005; Grazzani-Gavazzi and Ornaghi, 2011). Due to time restrictions, children did not complete a language ability task. Second, participants from the present study were Spanish from the same socioeconomic status. Thus, we have to be cautious when generalizing findings. Future research should examine the relation between parents' emotion talk and children's emotion understanding across different cultures and socioeconomic statuses. Third, the emotion task was a snapshot of parent-child interactions and focused only on verbal interactions. As mentioned previously, analysis of non-verbal cues may have demonstrated relations between fathers' behaviors and children's understanding.

## Conclusion

In conclusion, findings from the present study add to the existing knowledge of the relation between parents' emotion talk and children's emotion understanding. Findings indicated that whereas mothers' use of emotion labels during the play-related storytelling task predicted children's emotion understanding, fathers' emotion talk in this task did not predict children's emotion understanding after controlling for previous emotion understanding. These findings suggest that mothers and fathers may have different influences on children.

### Conflict of interest statement

The authors declare that the research was conducted in the absence of any commercial or financial relationships that could be construed as a potential conflict of interest.
